# Alkali-Activated Hybrid Cements Based on Fly Ash and Construction and Demolition Wastes Using Sodium Sulfate and Sodium Carbonate

**DOI:** 10.3390/molecules26247572

**Published:** 2021-12-14

**Authors:** William Valencia-Saavedra, Rafael Robayo-Salazar, Ruby Mejía de Gutiérrez

**Affiliations:** Composite Materials Group (GMC-CENM), Universidad del Valle, Cali 76001, Colombia; william.gustavo.valencia@correounivalle.edu.co (W.V.-S.); rafael.robayo@correounivalle.edu.co (R.R.-S.)

**Keywords:** fly ash, construction and demolition waste, alkaline activation, hybrid cements, alternative activators, sodium sulfate, sodium carbonate

## Abstract

This article demonstrates the possibility of producing alkali-activated hybrid cements based on fly ash (FA), and construction and demolition wastes (concrete waste, COW; ceramic waste, CEW; and masonry waste, MAW) using sodium sulfate (Na_2_SO_4_) (2–6%) and sodium carbonate (Na_2_CO_3_) (5–10%) as activators. From a mixture of COW, CEW, and MAW in equal proportions (33.33%), a new precursor called CDW was generated. The precursors were mixed with ordinary Portland cement (OPC) (10–30%). Curing of the materials was performed at room temperature (25 °C). The hybrid cements activated with Na_2_SO_4_ reached compressive strengths of up to 31 MPa at 28 days of curing, and the hybrid cements activated with Na_2_CO_3_ yielded compressive strengths of up to 22 MPa. Based on their mechanical performance, the optimal mixtures were selected: FA/30OPC-4%Na_2_SO_4_, CDW/30OPC-4%Na_2_SO_4_, FA/30OPC-10%Na_2_CO_3_, and CDW/30OPC-10%Na_2_CO_3_. At prolonged ages (180 days), these mixtures reached compressive strength values similar to those reported for pastes based on 100% OPC. A notable advantage is the reduction of the heat of the reaction, which can be reduced by up to 10 times relative to that reported for the hydration of Portland cement. These results show the feasibility of manufacturing alkaline-activated hybrid cements using alternative activators with a lower environmental impact.

## 1. Introduction

Alkali-activated cements and geopolymers are materials that, thanks to their good durability and mechanical, physical, and thermal properties, compete with materials such as Portland cement; red or kaolinitic clays; and many raw materials used to manufacture common or special ceramic materials, such as refractory ceramics, photoluminescent ceramics, and antibacterial ceramics. From an environmental point of view, this material can have a negative impact, mainly associated with the type of raw materials that compose it. For example, the use of silicates and hydroxides as activators, although they give rise to high mechanical strength and durability, are the activators that exhibit the highest CO_2_ load per kg of material produced [1,2]. However, in relation to the production process, the alkali-activated materials have advantages of low energy consumption and low generation of CO_2_ emissions, as its production is carried out at temperatures below 100 °C.

Considering that the key factor associated with the life cycle of a product is its positive contribution to climate change, the relevant indicator is the amount of greenhouse gases emitted into the environment, which is calculated by means of kilograms of CO_2_ equivalents (kg CO_2_ eq) of the product, or its so-called carbon footprint. The main relevant environmentally oriented approaches to reduce the kg CO_2_ eq associated with alkaline cements are the following: substituting alkaline activators such as sodium/potassium silicate and alkali hydroxides; use of supplementary raw materials such as industrial, agro-industrial, or municipal wastes, thus avoid exploiting soils; use of water from sources other than natural sources; and, in general, eliminating process stages that require a high energy expenditure (which is subsequently converted into CO_2_ emissions), such as calcination and sintering.

Alternatives that can replace silicate and sodium hydroxide include industrial solid activators such as sulfates and sodium carbonates, which have been investigated in recent years and can lead to more ecological, more sustainable, and less expensive cementitious systems [1,3]. However, in comparison with studies on other activators, studies that address activation with sodium sulfate and carbonate are very limited, and most of these have been carried out using slag as a precursor [1,4]. One possible reason is the low initial strength of this type of cement; Rashad et al. [5] report that the compressive strength of systems activated with sodium sulfate is lower than that achieved with other activators such as silicates and hydroxides. This behavior is corroborated by other researchers, who report that, although low resistances are obtained with this type of activator at early curing ages because of its lower pH, at prolonged ages, high resistances can be reached. In effect, the pH of sodium carbonate and sodium sulfate is approximately 12.6 and 8, respectively, while that of sodium silicate is 13.4 and that of sodium hydroxide is 13.8. The long-age resistant increase using sodium carbonate as an activator is attributed to the formation of carbonate compounds that contribute to densifying the microstructure [6]. It should be noted that other authors have investigated binary and ternary mixtures of these activators with NaOH, sodium silicate, and calcium hydroxide, and they report better performances both in terms of setting times and mechanical resistance, even at short ages [2,7,8,9]. Li and Sun [10] used Na_2_CO_3_ mixed with and without NaOH to activate blast furnace slag and a combination of slag and fly ash (FA), and the compressive strength of the systems activated with 10% Na_2_CO_3_ increased from 0 MPa at 3 days to 60 MPa at 28 days. Wang et al. [9] studied the reactivity of alkali-activated slag binders, using a mixture of calcium oxide and sodium carbonate as activators; the authors reported optimal proportions of 2.5% CaO and 5% Na_2_CO_3_ in order to obtain a much denser microstructure and higher compressive strength. Wang et al. [11], using a mixture of sodium silicate (49%), sodium aluminate (2%), and Na_2_CO_3_ (49%), obtained a longer setting time and lower heat of reaction in a FA/GBFS geopolymer. Bernal et al. [12] examined the mechanism of slag activation when using Na_2_CO_3_ and proposed that the activation is carried out in three different stages, starting with the dissolution of the slag and the formation of gaylussite and zeolite A on the first day. Then, the reaction can go through an extended induction period of 4 to 6 days with the conversion of gaylussite to CaCO_3_, the formation of hydrotalcite, and finally the precipitation of CASH gel [12]. Dung et al. [13] suggest incorporating reactive MgO to accelerate the reaction kinetics of Na_2_CO_3_ when activating GBFS, and they obtain a threefold increase in compressive strength at 28 days.

Regarding the alkaline activation of sources other than blast furnace slag (FA and CDW, among others), few studies have been performed. Donatello et al. [14] found that the hydration of a mixture of 80% FA and 20% OPC activated with 4% Na_2_CO_3_ and/or 4% Na_2_SO_4_ generates a mixture of CASH gels and NASH. In addition, calcite is formed in the case of Na_2_CO_3_ and ettringite when Na_2_SO_4_ is used as an activator, and the latter also accelerates the early hydration of alite. Dakhane et al. [15] evaluated the behavior of FA/OPC hybrid mixtures (75% FA) activated with Na_2_SO_4_ in proportions of 3% and 5% and reported that, at a curing age of 28 days, the resistance was only 30% and 40% of that obtained with the reference sample (100% OPC). Cristelo et al. [16] propose a hybrid cement based on FA, iron and steel slag, and OPC activated with Na_2_SO_4_, which presented compressive strength at 28 days of up to 40 MPa. The authors attribute the mechanical resistance to the greater dissolution of the precursor, as a consequence of the formation “in situ” of NaOH, and to the densification of the matrix by the formation and precipitation of ettringite. Villaquiran and Mejía de Gutiérrez [17] also evidenced the formation of ettringite in a CDW/OPC 80/20 paste activated with Na_2_SO_4_ and reported compressive strengths of up to 18 MPa at 28 days of curing at room temperature. Additonally, Portland concrete, with a high percentage of FA addition (60%) alkali-activated with sodium sulfate, showed better resistance performance, lower porosity, and reduced chloride penetration compared with the same type of concrete without activator [18]. Adesina et al. [1] recently reviewed the properties of cementitious materials activated with sodium sulfate, and stated that it is possible to achieve adequate strengths for structural applications by controlling the dosage of the activator, increasing the fineness of the precursor, or adding OPC or additives such as silica fume.

This article aims to investigate the compressive strength and microstructure of hybrid cements (10–30% OPC) based on different precursors such as FA, concrete waste (COW), and red ceramic waste (CEW) using sulfate (Na_2_SO_4_) and sodium carbonate (Na_2_CO_3_) as activators. Additionally, a precursor obtained from the mixture COW + CEW + masonry waste (MAW) in equal proportions (33.33%), which was called CDW, was investigated. It is expected that the results of this study will be the basis for the future use of FA and CDW as precursors for the manufacture of hybrid cements activated with sodium sulfate and carbonate, which will generate a lower environmental impact.

## 2. Materials and Experimental Methodology

As raw materials for the production of the alkaline activation hybrid systems, fly ash (FA), construction and demolition waste (COW, CEW, and MAW), and ordinary Portland cement (OPC) were used. The chemical composition of these materials, determined by X-ray fluorescence (XRF) using a MagiX-Pro PW-2440 spectrometer (Phillips PANalytical, Tollerton, USA), is given in Table 1. Approximately 88.98% of FA is composed of oxides of silica, alumina, and iron, and the unburned content (LOI) was 6.35%, so it could be classified as a type F, according to the ASTM C618 standard. The construction and demolition wastes are mainly composed of SiO_2_ and Al_2_O_3_, and COW has a high CaO content. Of the three materials, the highest unburned content (15.94%) was observed for COW. A new precursor called CDW was generated from the mixture COW + CEW + MAW in equal proportions (33.33%), and its composition is also included in Table 1.

**Figure 1:**
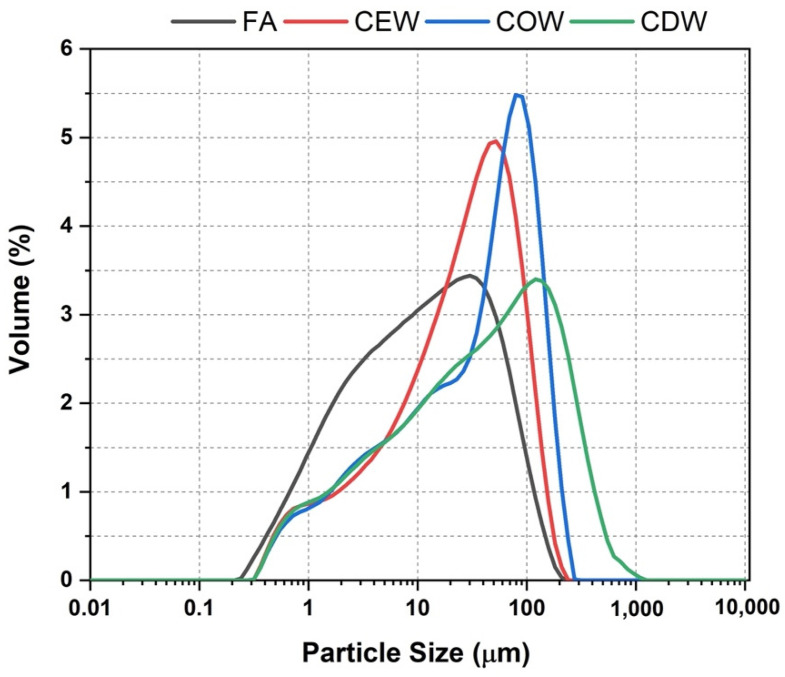
Particle size distribution of the precursors.

The mean particle size (D(4,3)) of the precursors FA, COW, CEW, and CDW, analyzed by laser granulometry with a Mastersizer-2000 (Malvern Instruments equipment, Malvern, UK), was 36 µm, 57 µm, 40 µm, and 75 µm, respectively. The particle size distributions of these materials are presented in Figure 1.

**Figure 2:**
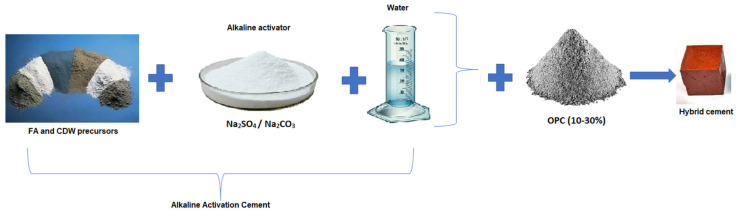
Schematic summary of the methodology developed to obtain alkaline activation materials based on FA and CDW.

The production of the alkaline activation materials from FA and the construction and demolition wastes (CEW, COW, and CDW) is summarized in Figure 2. The pastes were prepared in a Hobart mixer with a total mixing time of 5 min. The liquid/solid ratio (L/S) was 0.3. OPC was added in small proportions (10–30% by weight) to produce a “hybrid cement” (binder), which hardened and developed strength at room temperature. The fresh pastes were molded into 20 mm cubes and vibrated for 30 s on an electric vibrating table to remove the trapped air. Subsequently, the molds were covered with a polyethylene film and held in a laboratory environment for 24 h. After this time, the specimens were removed from the molds and placed in a final curing chamber (≈25 °C) with a relative humidity greater than 80% until reaching the test age.

To optimize the mixing proportions of the hybrid cement, the effects of the alkaline activator content (Na_2_SO_4_—2, 4, and 6%; Na_2_CO_3_—5, 7.5, and 10%) and OPC content (10, 20, and 30%) were evaluated by assessing the compressive strength of each paste at curing ages of 3, 7, 28, and 90 days. From these results, the optimal proportions were selected. In the produced pastes, the setting time, the heat of the reaction, and the mechanical resistance to compression were determined at ages of up to 180 days of curing. Pastes without alkaline activator and based on 100% OPC (type GU) were used as reference materials. The compressive strength was evaluated with an INSTRON 3369 universal testing machine, which has a capacity of 50 kN force, at a speed of 1 mm/min. For each mixture, a minimum of three specimens were tested. The setting time was determined according to the procedure described in the ASTM C191 standard (method B), and the heat evolution (during alkaline activation) and the total heat of reaction (68 h) were determined in an I-Cal 8000 isothermal calorimeter (Calmetrix, Needham, MA, USA).

Visual inspection of the samples was performed to observe the surface changes generated throughout the exposure time. The structural and mineralogical changes in each of the samples were evaluated using the following techniques:

- Fourier transfer infrared (FTIR) spectroscopy was performed using an R-100 spectrometer (Perkin Elmer, Shelton, CT, USA) in transmittance mode with a frequency between 4000 and 450 cm^−1^. The samples were evaluated using compressed KBr pellets.
- Scanning electron microscopy (SEM-EDS) was performed using a JSM 6490LV JEOL electron microscope (JEOL, Tokyo, Japan) with an acceleration voltage of 20 kV. The samples were evaluated in low-vacuum mode with a Link-Isis X-ray spectrometer (Oxford Instruments, Abingdon, UK) coupled to the microscope.

## 3. Results and Analysis

### 3.1. Characterization of Hybrid Cements Activated with Na_2_SO_4_ and Na_2_CO_3_

**Figure 3:**
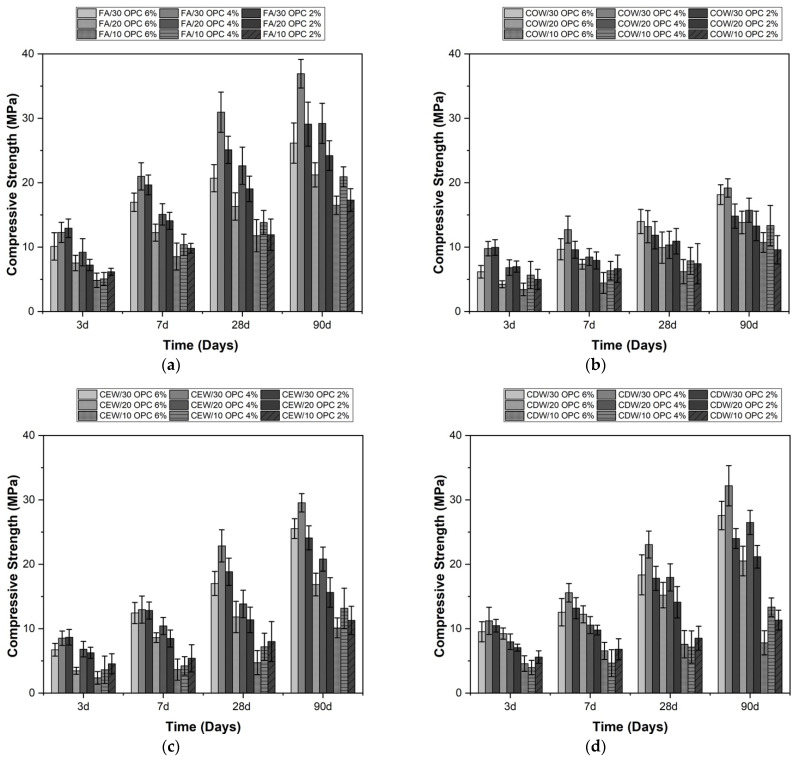
Compressive strength of alkaline-activated hybrid cements activated with Na_2_SO_4_. (**a**) FA/OPC, (**b**) COW/OPC, (**c**) CEW/OPC, and (**d**) CDW/OPC.

**Figure 4:**
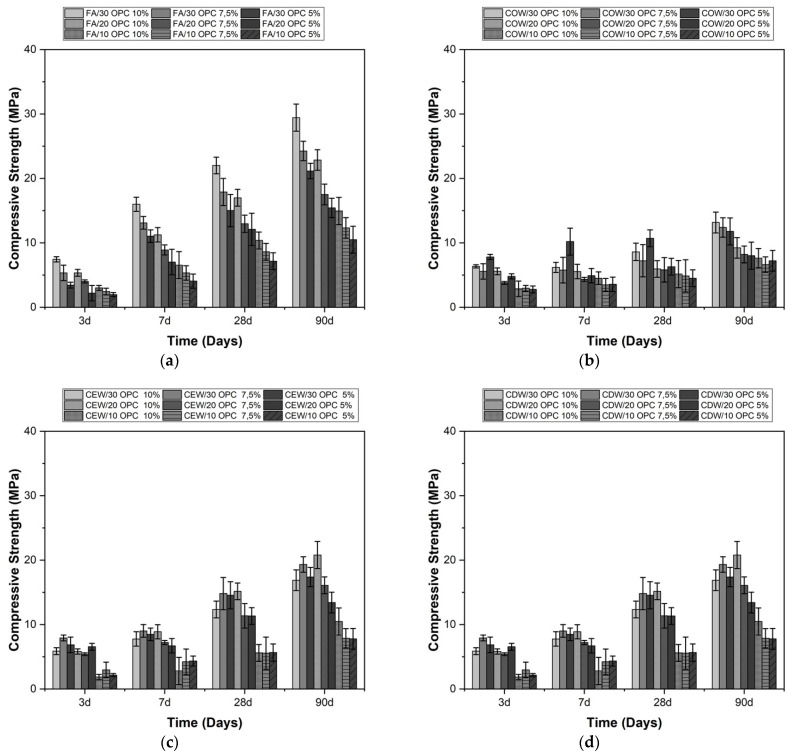
Compressive strength of alkaline-activated hybrid cements activated with Na_2_CO_3_. (**a**) FA/OPC, (**b**) COW/OPC, (**c**) CEW/OPC, and (**d**) CDW/OPC.

The effects of the Na_2_SO_4_/Na_2_CO_3_ and OPC contents on the compressive strength of the alkaline-activated hybrid systems can be observed in Figure 3 and Figure 4. In general, with higher cement contents (30%), higher compressive strength results were obtained at different curing ages, which coincides with the results reported by other authors [19].

In the case of hybrid systems activated with Na_2_SO_4_ (Figure 3), activation with 4% Na_2_SO_4_ produced a positive effect on the compressive strength, coinciding with the activator percentages recommended by other authors [5,20]. An increase in Na_2_SO_4_ content (6%) caused a decrease in the compressive strength of the hybrid systems at different curing ages, coinciding with the results reported by Zhao et al. [21]. This finding demonstrates the importance of studying the proportion of the activator in the mixture and determining the optimal content. In the case of sodium sulfate, the mechanical strength is attributable to the formation of ettringite, which contributes to greater densification of the material [15,19]. However, by increasing the proportion of the activator (>4%), the formation of an excess of ettringite likely causes microcracks, which can negatively affect the strength.

The compressive strengths of the hybrid systems activated with Na_2_CO_3_ are shown in Figure 4. The strength gain of the FA/OPC systems at early ages (3 and 7 days) was high, and a gradual increase was observed with prolonged curing time. Similarly, increasing the proportion of the activator from 5% to 10% significantly improved the strength of all mixtures. This result indicates that increases in alkali content (Na_2_O) play a vital role in the development of mechanical strength. The improvement can be attributed to the fact that a higher Na_2_O content increases the pH of the system, which increases the dissolution of reactive SiO_2_ and Al_2_O_3_ [22]. This greater dissolution in turn increases the generation of reaction products and, consequently, increases resistance. In this study, compressive strengths of up to 35 MPa were reached at 90 days of curing; these results agree with those reported by Abdalqader et al. [22] and Li and Sun [10]. The inclusion of up to 30% OPC in the systems showed a satisfactory increase in the compressive strength, coinciding with that observed in the systems activated with sodium sulfate.

In general, from the results reported in Figure 3 and Figure 4, the hybrid cementitious systems with a higher OPC content (30%) reached strengths of up to 37 MPa when subjected to alkaline activation with Na_2_SO_4_ (4%) and 30 MPa when activated with Na_2_CO_3_ (10%). These results correspond to those obtained at 90 days of curing. FA/OPC and CDW/OPC stood out among the different systems evaluated. 

The increase in compressive strength with the curing age of the FA/OPC mixtures activated with Na_2_SO_4_ and Na_2_CO_3_ (Figure 3a and Figure 4a) is higher than that of COW/OPC, CEW/OPC, and CDW/OPC. This difference can be attributed to the smaller particle size of FA and the higher proportion of amorphous phase present and generally coincides with the results reported by other studies [19].

The hybrid CDW/OPC systems activated with sodium sulfate (Figure 3d) demonstrated better mechanical performance than that observed for COW and CEW. The good behavior of CDW/OPC hybrid systems activated with Na_2_SO_4_ coincides with that reported by other researchers [17], who demonstrated that, the higher the OPC content, the greater the resistance. Furthermore, they report optimal percentages of Na_2_SO_4_ between 1 and 5%. 

Therefore, for the following phases of the study, FA/30OPC-4%Na_2_SO_4_, CDW/30OPC-4%Na_2_SO_4_, FA/30OPC-10%Na_2_CO_3_, and CDW/30OPC-10%Na_2_CO_3_, were selected as optimal mixtures.

**Figure 5:**
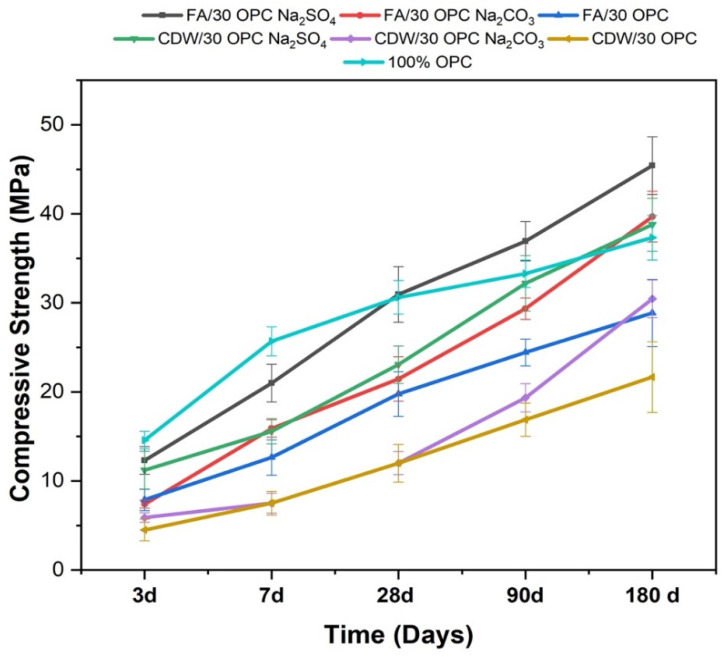
Evolution of the compressive strength of alkaline-activated hybrid cements based on FA and CDW (optimal mixtures): comparison with a paste based on the optimal hybrid cement without activation and 100% OPC (reference mixtures).

Figure 5 shows the evolution of the compressive strength of the optimal cementitious materials mentioned above as a function of curing time (1–180 days) in comparison with the reference pastes, which correspond to the same type of mixture without activator and were based on 100% OPC. All the pastes tended to exhibit increased resistance with the evolution of curing time, highlighting that, in general, the mechanical performance of the OPC paste was superior to that of the pastes of the different alkaline-activated hybrid systems and the pastes without activation at short curing ages. However, it should be noted that this difference decreased from 28 to 180 days, a period in which the alkaline activation pastes FA/30OPC-4%Na_2_SO_4_, CDW/30OPC-4%Na_2_SO_4_, and FA/30OPC-10%Na_2_CO_3_ presented a higher strength gain than 100% OPC paste. The FA/30OPC-4%Na_2_SO_4_ paste exhibited the best performance, with a compressive strength value of 45 MPa, 22% better than the values for OPC pastes and systems without alkaline activation. This behavior agrees with that reported by Rashad et al. [5], Joseph et al. [19], Velandia et al. [23], and Zhao et al. [21], who obtained similar resistance increases for alkaline activation systems activated with sodium sulfate at long ages. The increases in resistance for the different alkaline activation systems are related to the densification of the matrix and the greater formation of “hydrated sodium-calcium aluminosilicates” or gels, (N,C)-ASH, CASH, carbonates, and ettringite with prolonged curing time [14,22]. The samples activated with Na_2_CO_3_ show slow compressive strength development. The strength at early ages is low; however, at later ages, an increase is observed (Figure 5). This increase is associated with the formation of calcium carbonate, which densifies the structure [4].

### 3.2. Reaction Monitoring and Characterization of Reaction Products

**Figure 6:**
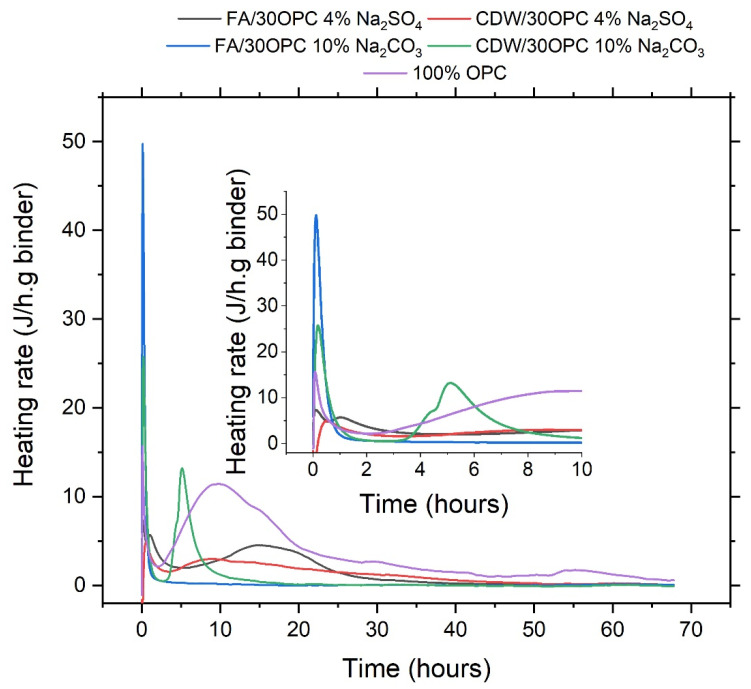
Isothermal calorimetry: reaction kinetics of hybrid cements based on FA and CDW (optimal mixtures).

Figure 6 presents the calorimetric curves of the alkaline activation systems FA/30OPC-4%Na_2_SO_4_, FA/30OPC-10%Na_2_CO_3_, CDW/30OPC-4%Na_2_SO_4_, and CDW/30OPC-10%Na_2_CO_3_, compared to 100% OPC paste (100% OPC + H_2_O). 

For the different alkaline-activated hybrid systems, there is a first peak that, in general, occurs in less than 10 min. The intensity of the peak varies depending on the activation conditions, but the maximum value for the systems activated with Na_2_SO_4_ is 8 J/gh, and that for the systems activated with Na_2_CO_3_ is 50 J/gh. For the systems activated with Na_2_SO_4_, the acceleration phase (~5–15 h, depending on the system) occurs at a longer time than that observed for OPC hydration (~2–10 h), mainly owing to the dilution effect [15]. For the FA/30OPC-10%Na_2_CO_3_ system, a single peak is observed, which could be related to the greater alkalinity of the system and the greater reactivity of FA, as a result of which the preinduction, induction, and acceleration peaks are not separately identifiable because dissolution and precipitation reactions develop simultaneously for this system [24]. For the CDW/30OPC-Na_2_SO_4_ system, the acceleration peak is observed, which is attributed to the formation of reaction products of CASH/CSH and (Ca, Na)-ASH. The heat evolution rate for the CDW/30OPC-10%Na_2_CO_3_ system during the acceleration period is 13 J/gh, which is higher than that for the systems activated with Na_2_SO_4_. This result could be related to the greater alkalinity of the medium. Similarly, a reduction in the time of the acceleration peak is observed, which could be related to the greater reactivity of the system.

The total heat released for the hybrid systems FA/30OPC-4%Na_2_SO_4_, FA/30OPC-10%Na_2_CO_3_, CDW/30OPC-4%Na_2_SO_4_, and CDW/30OPC-10%Na_2_CO_3_ is compared with that of 100% OPC in Table 2. Alkaline activation systems activated with Na_2_SO_4_ are much more reactive than hybrid systems activated with Na_2_CO_3_, although the total heat released for the alkaline activation systems is much lower than that of 100% OPC. In general, the greatest heat release in these systems occurs in the first hours and is rapidly attenuated or stabilized. This behavior differs from the hydration process of OPC, where the release of heat increases over time. Table 2 shows that the systems activated with Na_2_CO_3_ present a notable reduction in the initial and final setting times compared with those observed for the hybrid system activated with Na_2_SO_4_. This result could be related to the greater alkalinity of Na_2_CO_3_ (pH = 12.16) than Na_2_SO_4_ (pH = ~8), which accelerates the alkaline activation reactions. The final setting time for the FA/30OPC-10%Na_2_CO_3_ system is reduced by 20% when compared with the FA/30OPC-4%Na_2_SO_4_ system. In the case of the CDW/hybrid systems, 30OPC-10%Na_2_CO_3_ shows a reduction of 80% when compared with CDW/30OPC-4%Na_2_SO_4_.

**Figure 7:**
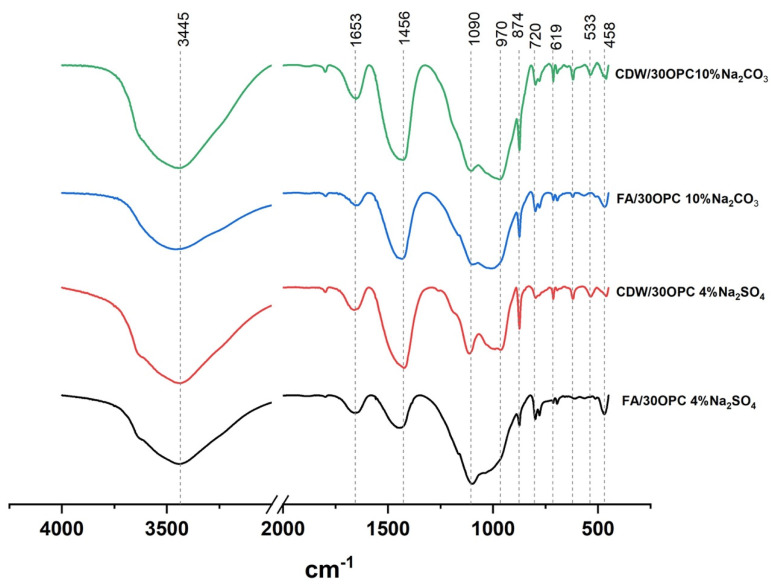
Infrared (FTIR) spectra of the alkaline activation hybrid systems.

The FTIR results for the hybrid systems FA/30OPC-4%Na_2_SO_4_, FA/30OPC-10%Na_2_CO_3_, CDW/30OPC-4%Na_2_SO_4_, and CDW/30OPC-10%Na_2_CO_3_ at 180 days of curing are shown in Figure 7. The broad band centered at ±3445 cm^−1^ is associated with stretching of O-H bonds, which indicates the presence of adsorbed water [25,26]. The bands located at 1653 cm^−1^ in the materials activated with Na_2_SO_4_ and Na_2_CO_3_ correspond to the bending vibration of the water molecules linked to the inorganic structure and the presence of -OH groups [17,25,27,28]. A greater intensity of this band has been associated with greater amounts of reaction products of CSH, CASH, and (N, C)-ASH types [17]. 

In the systems activated with Na_2_SO_4_ and Na_2_CO_3_, two bands appear at 1090 and ~970 cm^−1^, and these bands indicate the substitution of SiO_4_ species by AlO_4_ [27,28] due to the formation of new products; CSH gel (normally detected at approximately 970 cm^−1^) [28] and gels such as C-(A)-SH have been associated with peaks at wavenumbers of 1090, 1085, and 1005 cm^−1^ [17]. In addition, this main band is wide and could indicate the coexistence of several types of gels, such as C-(A)-SH, CSH, and (N),C-(A)-SH [29]. Calcium carbonates are present in different alkaline activation systems, as indicated by the band at approximately 1430 cm^−1^, which is associated with the asymmetric tension of O-C-O bonds in CO_3_^−2^ groups [30], and a band at approximately 850 cm^−1^ that indicates the stretching vibration of C-O groups [31,32,33]. The alkaline activation systems FA/30OPC-10%Na_2_CO_3_ and CDW/30OPC-10%Na_2_CO_3_ exhibit greater intensity than that observed for the systems activated with Na_2_SO_4_. The band at approximately 720 cm^−1^ is attributed to the presence of mullite present in the raw materials (FA and CDW) [34]. The band identified at 1090 cm^−1^ for the samples activated with sodium sulfate indicates the presence of anhydrous calcium sulfate (CaSO_4_); other bands associated with the presence of semihydrated calcium sulfates located at 660 cm^−1^ [26] were not observed, possibly owing to the overlap of the bands associated with aluminate species.

### 3.3. Microstructural Characterization of Alkali-Activated Hybrid Cements by Scanning Electron Microscopy (SEM-EDS)

**Figure 8:**
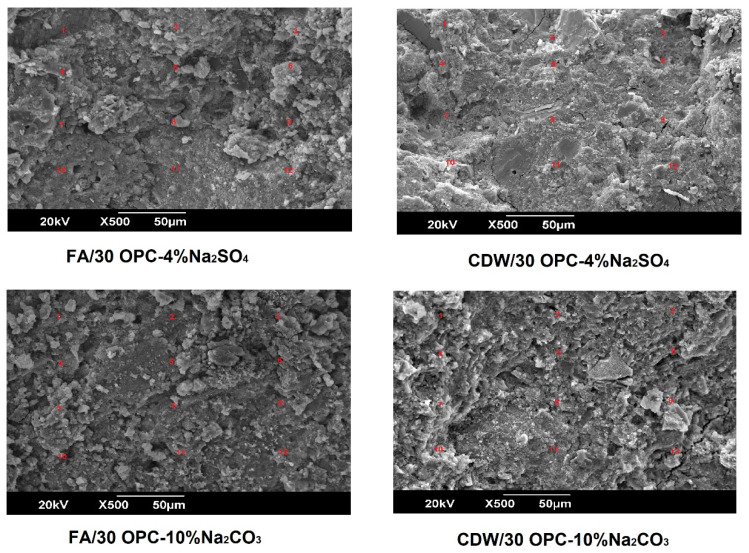
Microstructure of the hybrid alkaline activation systems.

**Figure 9:**
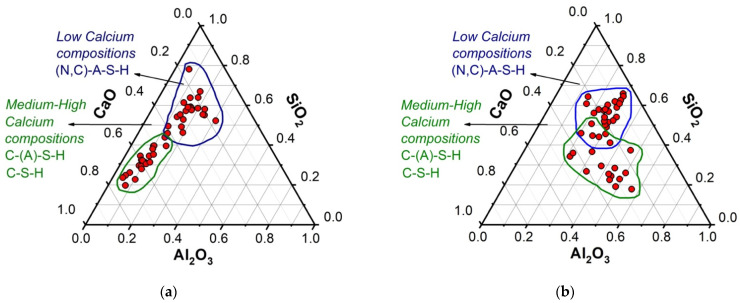
Microstructural compositional analysis (SEM-EDS) of the alkali-activated hybrid cements: (**a**) Na_2_SO_4_ and (**b**) Na_2_CO_3_.

Figure 8 presents the SEM-EDS microstructural analysis of the FA/30OPC-4%Na_2_SO_4_, FA/30OPC-10%Na_2_CO_3_, CDW/30OPC-4%Na_2_SO_4_, and CDW/30OPC-10%Na_2_CO_3_ hybrid systems at 180 days of curing. Through this technique, it was possible to study the elemental chemical composition of the different systems and create ternary diagrams of SiO_2_-Al_2_O_3_-CaO (Figure 9). These diagrams represent a total of 24 EDS points analyzed in each of the systems (FA/30OPC-4%Na_2_SO_4_, FA/30OPC-10%Na_2_CO_3_, CDW/30OPC-4%Na_2_SO_4_, and CDW/30OPC-10%Na_2_CO_3_). The results for the different hybrid alkaline activation systems are grouped into “low calcium compositions” and “medium-high calcium compositions”. Some authors have correlated representative areas of the SiO_2_-Al_2_O_3_-CaO ternary diagram with reaction product type, (N,C)-ASH (poor in Ca^2+^), CSH, and CASH (rich in Ca^2+^) for alkali-activated materials and hybrid cements based on other precursors, such as FA, GBFS, and/or natural pozzolans [30,35,36,37,38].

**Figure 10:**
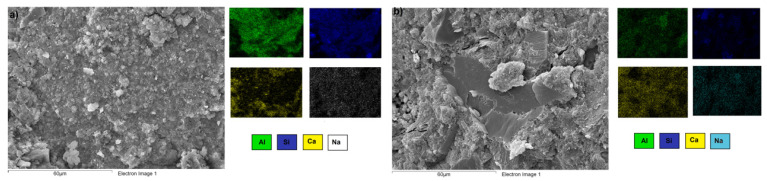
EDS analysis of the hybrid systems activated with Na_2_SO_4_. (**a**) FA/30OPC-4%Na_2_SO_4_ and (**b**) CDW/30OPC-4%Na_2_SO_4_.

**Figure 11:**
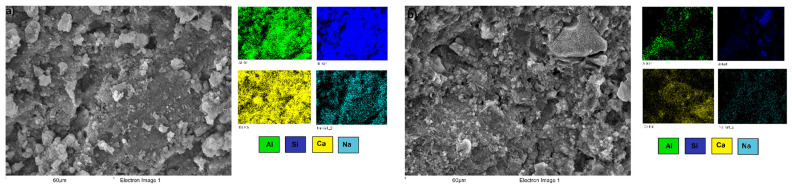
EDS analysis of the hybrid systems activated with Na_2_CO_3_. (**a**) FA/30OPC-10%Na_2_CO_3_ and (**b**) CDW/30OPC-10%Na_2_CO_3_.

Elemental EDS color mapping was performed on a specific area of the FA/30OPC-4%Na_2_SO_4_, FA/30OPC-10%Na_2_CO_3_, CDW/30OPC-4%Na_2_SO_4_, and CDW/30OPC-10%Na_2_CO_3_ hybrid systems (Figure 10 displays systems activated with Na_2_SO_4,_ and Figure 11 displays systems activated with Na_2_CO_3_). The results showed a homogeneous distribution of silica (Si: blue), alumina (Al: green), sodium (Na: turquoise), and calcium (Ca: yellow) in the pastes, corroborating the formation and/or coexistence of NASH, CSH, CASH, and (N,C)-ASH gels resulting from chemical interactions between the precursors (FA and CDW), OPC, and the alkaline activator (Na_2_SO_4_ or Na_2_CO_3_).

## 4. Conclusions

In this study, the effects of Na_2_SO_4_ and Na_2_CO_3_ on the alkaline activation of hybrid cements based on fly ash and construction and demolition wastes were investigated. The proportions of activators and OPC in the mixtures were studied, and the compressive strength at ages of up to 180 days, the hydration process, and the microstructure were evaluated. From the results, the following conclusions can be drawn.

FA has greater reactivity than construction and demolition waste in the presence of alkaline media (Na_2_SO_4_ and Na_2_CO_3_), where an optimal proportion of activating solution yields alkaline-activated materials with a mechanical strength of 25 MPa for hybrid cements based on FA with Na_2_CO_3_ activation and 32 MPa for systems activated with Na_2_SO_4_ at 28 days of curing, room temperature (24 °C), and relative humidity >90%.

In hybrid alkaline activation systems based on construction and demolition waste, better performance was obtained in those based on the mixture of CDW/OPC, which may be related to the positive synergistic effect promoted by the combination of COW powder, CEW, and MAW.

In general, the optimal percentage of Na_2_SO_4_ activator was 4%, and in the case of the Na_2_CO_3_ activator, the best mechanical behavior was obtained when using 10%. Regarding the OPC content, the greatest strength increases were achieved with 30% OPC.

The isothermal calorimetry results showed that the heat of the reaction of the hybrid cements activated with Na_2_SO_4_ is higher than that of the hybrid cements activated with Na_2_CO_3._ This behavior indicates that the systems activated with Na_2_SO_4_ present a greater degree of reaction. However, in general, the heat of reaction of the activated systems is lower than the total heat released in the hydration of the 100% OPC pastes. The SEM-EDS microstructural analysis performed on the hybrid pastes FA/30OPC-4%Na_2_SO_4_, FA/30OPC-10%Na_2_CO_3_, CDW/30OPC-4%Na_2_SO_4_, and CDW/30OPC-10%Na_2_CO_3_ demonstrated the formation of reaction products of the type (N,C)-ASH (low in Ca^2+^), CSH, and CASH (rich in Ca^2+^) in the cementing phase.

In general, these results indicate the viability of producing cementitious pastes activated with sodium sulfate and sodium carbonate from precursors such as FA and CDW, leading to products with greater technological and potentially environmental sustainability.

## Figures and Tables

**Table 1:** Chemical composition of the materials used.

Material		SiO_2_	Al_2_O_3_	Fe_2_O_3_	CaO	MgO	Na_2_O	SO_3_	TiO_2_	LOI
Precursors	FA	59.03	23.97	5.98	0.74	0.31	0.19	0.55	0.95	6.35
	COW	36.13	8.33	6.78	28.66	1.85	0.64	1.16	0.35	15.94
	CEW	59.63	16.10	5.48	9.79	0.77	0.45	0.35	0.72	4,07
	MAW	59.04	18.42	7.76	5.37	2.39	1.05	0.21	0.80	2.92
	CDW	51.60	14.28	6.67	14.61	1.67	0.71	0.57	0.62	7.64
OPC		19.13	4.42	4.32	57.70	1.60	-	2.32	-	9.78

**Table 2:** Heat of reaction and setting time.

Sample	Initial Setting Time (min)	Final Setting Time (min)	Heat of Reaction (J/g)
FA/30OPC-4%Na_2_SO_4_	88	146	94.03
CDW/30OPC-4%Na_2_SO_4_	350	570	80.13
FA/30OPC-10%Na_2_CO_3_	90	116	24.90
CDW/30OPC-10%Na_2_CO_3_	92	115	53.70
100% OPC	-	-	234.62
